# Risk Factor Analysis and Prediction of Para‐Aortic Lymph Node Metastases in Locally Advanced Cervical Cancer

**DOI:** 10.1002/cam4.70492

**Published:** 2024-12-16

**Authors:** Tinglu Wang, Jinchen Wei, Li Jiang, Lulu Huang, Tingting Huang, Shanshan Ma, Qiufeng Huang, Yong Zhang, Fang Wu

**Affiliations:** ^1^ Department of Radiation Oncology The First Affiliated Hospital of Guangxi Medical University Nanning Guangxi China

**Keywords:** cervical cancer, lymph node metastases, nomogram, PET/CT, risk factors

## Abstract

**Background and Purpose:**

The indications of prophylactic extended‐field radiotherapy (EFRT) remain uncertain. This study aims to identify the risk factors for para‐aortic lymph node (PALN) metastases in locally advanced cervical cancer (LACC) and determine which part of patients may benefit from prophylactic EFRT.

**Materials and Methods:**

Between January 2015 and July 2023, a single‐center retrospective analysis was performed on patients with stages IB3 and IIA2‐IVA cervical cancer. Lymph node involvement was assessed using positron emission tomography/computed tomography (PET/CT). Risk factors were evaluated by logistic regression. A prediction nomogram model was developed and validated.

**Results:**

Among 329 patients, 64 (19.5%) had PALN metastases. Univariate analysis indicated that tumor size > 5.3 cm, tumor maximum standardized uptake value (SUVmax) > 9.8, bilateral pelvic lymph node (PLN) metastases, the number of positive PLNs ≥ 3, and T3–T4 stages were related to PALN metastases. After multivariate logistic analysis, it was found that tumor size > 5.3 cm (odds ratio [OR] = 3.129, 95% confidence interval [CI] = 1.536–6.374, *p* = 0.002), and the number of positive PLNs ≥ 3 (OR = 11.260, 95% CI = 3.506–36.158, *p* < 0.001) were independent risk factors. The C‐index of the nomogram was 0.886 (95% CI = 0.844–0.927). The calibration plot showed that the nomogram was well‐fitted. Decision curve analysis (DCA) exhibited excellent clinical utility.

**Conclusion:**

Tumor size > 5.3 cm and the number of positive PLNs ≥ 3 are independent risk factors of PALN metastases. The nomogram shows pretty good accuracy, which may provide a valuable reference for guiding patients who are very likely to develop PALN metastases to receive prophylactic EFRT.

## 1 Introduction

Cervical cancer, which is ranked as the world's fourth most common cancer among women, poses a significant public health challenge, with 604,000 new cases and 342,000 deaths reported in 2020 [1]. Early‐stage cervical cancer has a 5‐year relative survival rate of 91.2%, while regionally advanced disease has a significantly lower 5‐year survival rate of 59.8% [2]. For locally advanced cervical cancer (LACC) patients, concurrent chemoradiotherapy (CCRT) is considered the standard treatment and achieves an overall survival of 71.0%–77.6% [3, 4]. For patients with stage IIIC2 tumors, including para‐aortic lymph node (PALN) metastases, however, the survival rate significantly decreases to 37.5% [5]. Consequently, the prognosis of cervical cancer becomes particularly grim in the presence of PALN metastases.

In the management of LACC, the recurrence of PALNs is one of the most common sites of treatment failure [6]. In an EMBRACE study cohort [7], 152 of 1388 LACC patients (11%) developed nodal failure. Additionally, 68% of nodal failures occurred in the para‐aortic region. Strikingly, 40% of nodal failures extended beyond treatment targets. The para‐aortic region is not covered in standard pelvic RT fields. According to Nomden et al. [7], 12% of patients with positive pelvic lymph nodes (PLNs) but negative PALNs developed PALN failure after receiving CCRT and image‐guided brachytherapy. This indicates that standard radical pelvic RT is likely to be inadequate for high‐risk patients with occult cancer within PALNs.

Extended‐field radiotherapy (EFRT) expands the whole pelvis external‐beam RT to include PALNs as well. Research suggests that prophylactic EFRT can lower the rates of distant and para‐aortic failures, which highlights its potential effectiveness in this context [8, 9]. Specifically, EFRT may treat occult PALN metastases that have not been detected by traditional diagnostic techniques. Nevertheless, the application of RT in the para‐aortic region risks increasing severe chronic toxicity [9, 10, 11]. The indications of prophylactic EFRT are uncertain at present. The identification of patients who are most likely to benefit from this treatment strategy, particularly those at an increased risk of PALN metastases, is crucial for optimizing the balance between therapeutic gains and potential risks.

The status of PALN metastases in LACC can be determined by pathology and/or imaging [12]. A prospective multicenter study showed that no difference was found between laparoscopic surgical and clinical staging in LACC [13]. For patients with LACC, where CCRT is the best treatment option, radiological staging can be conducive to avoiding unnecessary surgery, which allows patients to undergo initial tumor treatment as soon as possible. In the comparison of various imaging methods for the detection of lymphadenopathy, positron emission tomography/computed tomography (PET/CT) appeared as the most sensitive examination [14]. To be specific, PET or PET/CT was highest in the combined sensitivity (82%) and specificity (95%). By comparison, magnetic resonance imaging (MRI) and CT had a sensitivity of 56% and 50%, and a specificity of 91% and 92%, respectively. Accurately determining the status of PALN metastases is essential for optimizing treatment strategies in LACC.

In the present study, the status of lymph node metastasis in LACC patients was evaluated using PET/CT, and risk factors for PALN metastases were identified. Additionally, patients who are most likely to benefit from prophylactic EFRT were determined.

## 2 Material and Methods

### 2.1 Patient Data

A single‐center retrospective clinical study was performed on LACC patients to identify the risk factors of PALN metastases and create a nomogram for predicting PALN metastases. Patients diagnosed with LACC at the First Affiliated Hospital of Guangxi Medical University in China were retrospectively screened from January 2015 to July 2023. Inclusion criteria were listed below: (1) Patients diagnosed with pathologically confirmed cervical cancer; (2) Patients with disease stages classified as IB3 or IIA2 to IVA following the 2018 Federation International of Gynecology and Obstetrics (FIGO) staging system; (3) Patients who accepted PET/CT and pelvic MRI or CT before initial treatment. Patients were excluded if they had any of the following: (1) a history of other malignant tumors; (2) prior pelvic or para‐aortic lymph node dissection; (3) previous treatment for cervical cancer, with a current diagnosis of recurrence and metastasis. This study was approved by the Ethics Committee of the First Affiliated Hospital of Guangxi Medical University (protocol 2023‐E323‐01). Informed consent was deemed unnecessary due to the anonymization of the data utilized.

In total, 329 LACC patients were enrolled, and clinical data were obtained from entirely computerized medical records. The following factors were accounted as the risk factors of PALN metastases: age, human papillomavirus (HPV) infection status, tumor histological type, tumor size and stage, tumor maximum standardized uptake value (SUVmax), bilateral PLN metastases, the number of positive PLNs, as well as vaginal and parametrial involvement.

### 2.2 Evaluation

In this study, pelvic MRI or CT scans were used to measure the size of the maximum tumor diameter, while vaginal and parametrial involvement was assessed by gynecological examination. The presence of PALN, PLN, and other lymph node metastases was determined using PET/CT scans at the time of cervical cancer diagnosis. Lymph nodes with SUVmax greater than 2.5 g/mL were classified as positive for metastasis. Furthermore, patients who presented with lymph node metastases on the two sides of the pelvis were identified to have bilateral PLN metastases.

To facilitate tumor characterization, patients were classified into two categories based on tumor size (≤ and > 5.3 cm) and SUVmax (≤ and > 9.8), with cut‐off values derived from receiver operator characteristic (ROC) curve analysis. Tumors were staged as per the 9th edition of the American Joint Committee on Cancer (AJCC) classification system. Images were analyzed and processed independently by two attending radiation oncologists. Differences of opinions were resolved by discussion.

### 2.3 Treatment

The treatment plans for patients were developed according to NCCN guidelines. All patients underwent radical radiotherapy, and the delineation of the external beam radiotherapy (EBRT) target volumes was performed based on the RTOG contour consensus. The gross tumor volume (GTV) included the tumor identified by imaging or clinical examination, such as cervical lesions, involved parametrium, and infiltrated tissues.

PLN metastases were contoured as GTVnd‐1, and PALN metastases as GTVnd‐2. The clinical target volume (CTV) included the cervix, uterus, parametrium, and a portion of the vagina (the upper half was included if the vagina was not involved or only minimally involved; the upper two‐thirds were included if the upper third of the vagina was involved; the entire vagina was included if more extensive vaginal involvement was present), and the pelvic lymph node region. In cases of the lower third vaginal involvement, bilateral inguinal lymph nodes were also included. When PALN metastases were present, the para‐aortic region was included in the CTV. After completion of EBRT, high‐dose‐rate (HDR) brachytherapy was administered. The prescribed dose to the planning target volume (PTV) for EBRT was 45–50 Gy in 1.8–2.0 Gy fractions, with a dose of 60 Gy for PGTVnd. The total dose, including both external beam and intracavitary brachytherapy, was 75–90 Gy. During EBRT, patients received concurrent weekly cisplatin (40 mg/m^2^) for 5–6 cycles, or an alternative 5‐fluorouracil‐based regimen for those who were unable to tolerate platinum‐based chemotherapy.

### 2.4 Statistical Analysis

The statistical package R (version 4.2.1.) was utilized for plotting and statistical analysis. The predictive values of tumor size and SUVmax for PALN metastases were analyzed by ROC curve analysis, and cut‐off values were determined by Youden's index (sensitivity + specificity—1). Univariable and multivariable logistic regressions were evaluated for factors associated with PALN metastases. Odds ratios (ORs) along with 95% confidence intervals (CIs) were computed. The accuracy of these tests was assessed by calculating the areas under the ROC curve (AUCs). All the *p*‐values derived from two‐sided tests were considered to show statistical significance at *p* < 0.05.

Following the logistic regression analysis, a prediction nomogram was constructed through covariates demonstrating a *p*‐value < 0.1 in univariate analysis. Three approaches were adopted to appraise the efficacy of the nomograph model, namely discrimination (via the C‐index or concordance index), calibration (via a calibration plot), and clinical utility (via decision curve analysis, DCA). The internal validity of the model was evaluated by calculating C‐index with 500 bootstrap samples. The calibration plot showed the predicted probabilities from the nomogram versus actual probabilities. DCA was used to determine the net benefit across a variety of clinically relevant risk thresholds. Moreover, the ROC curve was employed to assess the predictive performance of the model.

## 3 Results

### 3.1 ROC Curve Analysis

The ROC curve showed that the best threshold for predicting PALN metastasis was 5.35, the area under the curve was 0.719 (95% CI: 0.650–0.787), the sensitivity was 71.9%, and the specificity was 67.9%. The best cut‐off value of tumor SUVmax for predicting PALN metastasis was 9.85, the area under the curve was 0.560 (95% CI: 0.487–0.634), the sensitivity was 82.8%, and the specificity was 33.6%. Combined with the optimal cut‐off value and clinical practice, the tumor length was divided into ≤ 5.3 cm and > 5.3 cm, and the SUVmax of tumor was divided into ≤ 9.8 and > 9.8.

### 3.2 Patient Characteristics

Between January 2015 and July 2023, 329 patients were encompassed in this study. Among them, 64 (19.5%) reported PALN metastases based on PET/CT, whereas 265 (80.5%) had negative PALNs. The characteristics of patients are displayed in Table 1.

**TABLE 1:** Characteristics of patients.

Characteristics	All (*n* = 329)	Negative PALN (*n* = 265)	Positive PALN (*n* = 64)	*p*
Age, years old, mean ± SD	55.08 ± 9.69	55.05 ± 9.63	55.20 ± 10.02	0.907
HPV infection, *n* (%)				
Positive	129 (39.2)	103 (38.9)	26 (40.6)	0.548
Negative	13 (4.0)	12 (4.5)	1 (1.6)	
Not know	187 (56.8)	150 (56.6)	37 (57.8)	
Histological type, *n* (%)				
SCC	286 (86.9)	231 (87.2)	55 (85.9)	0.793
Non‐SCC	43 (13.1)	34 (12.8)	9 (14.1)	
Tumor size, cm, mean ± SD	5.08 ± 1.72	4.82 ± 1.64	6.15 ± 1.66	< 0.001
Tumor size, cm, *n* (%)				
≤ 5.3	131 (39.8)	85 (32.1)	46 (71.9)	< 0.001
> 5.3	198 (60.2)	180 (67.9)	18 (28.1)	
Tumor SUVmax, mean ± SD	13.87 ± 7.19	13.74 ± 7.38	14.42 ± 6.35	0.497
Tumor SUVmax, *n* (%)				
≤ 9.8	229 (69.6)	176 (66.4)	53 (82.8)	0.010
> 9.8	100 (30.4)	89 (33.6)	11 (17.2)	
Bilateral PLN metastases, *n* (%)				
No	209 (63.5)	199 (75.1)	10 (15.6)	< 0.001
Yes	120 (36.5)	66 (24.9)	54 (84.4)	
The number of positive PLN, *n* (%)				
< 3	202 (61.4)	196 (74.0)	6 (9.4)	< 0.001
≥ 3	127 (38.6)	69 (26.0)	58 (90.6)	
Vaginal involvement, *n* (%)				
No	127 (38.6)	98 (37.0)	29 (45.3)	0.219
Yes	202 (61.4)	167 (63.0)	35 (54.7)	
Parametrial involvement, *n* (%)				
No	113 (34.3)	90 (34.0)	23 (35.9)	0.765
Yes	216 (65.7)	175 (66.0)	41 (64.1)	
Tumor stage, *n* (%)				
T1b–T2	232 (70.5)	195 (73.6)	37 (57.8)	0.013
T3–T4	97 (29.5)	70 (26.4)	27 (42.2)	

Abbreviations: HPV = human papillomavirus, Non‐SCC = non‐squamous cell carcinoma, PALN = para‐aortic lymph node, PLN = pelvic lymph node, SCC = squamous cell carcinoma, SD = standard deviation, SUVmax = maximum standardized uptake value.

All the patients were 55.08 ± 9.69 years old on average. HPV infection status was not tested before treatment in over half of patients (*n* = 187, 56.8%). The predominant histological type was squamous cell carcinoma which comprised 286 cases (86.9%). Among 43 patients with nonsquamous cell carcinoma, 34 reported adenocarcinoma. The mean tumor size and SUVmax were 5.08 ± 1.72 cm and 13.87 ± 7.19, respectively. The patient cohort was categorized into two groups according to the status of PALNs: negative (*n* = 265) and positive PALN groups (*n* = 64). Among 86 patients with tumor sizes less than or equal to 4.0 cm, seven (8.1%) developed PALN metastases. None of the eight patients with small tumors (≤ 2.0 cm) developed PALN metastases. Among 64 patients with PALN metastases, one (1.6%) had a negative for metastasis; two (3.1%) had only one PLN metastasis; three (4.7%) had two PLN metastases; the other 58 (90.6%) had at least three PLN metastases.

### 3.3 Risk Factors Predicting the PALN Metastases of LACC Patients

As presented in Table 1, univariate analysis showed that age, HPV infection, histological type, as well as vaginal and parametrial involvement showed no differences between the two groups. However, the positive PALN group showed an association with tumor size > 5.3 cm (*p* < 0.001), tumor SUVmax > 9.8 (*p* = 0.010), bilateral PLN metastases (*p* < 0.001), the number of positive PLNs ≥ 3 (*p* < 0.001), and T3–T4 stages (*p* = 0.013).

As illustrated in Table 2, the univariate logistic analysis indicated that tumor size > 5.3 cm, tumor SUVmax > 9.8, bilateral PLN metastases, the number of positive PLNs ≥ 3 and T3–T4 stages were notably linked to PALN metastases (*p* < 0.001, *p* = 0.012, *p* < 0.001, *p* < 0.001, and *p* < 0.014, respectively). Multivariate logistic regression analysis revealed that tumor size > 5.3 cm (OR = 3.129, 95% CI = 1.536–6.374, *p* = 0.002) and the number of positive PLNs ≥ 3 (OR = 11.260, 95% CI = 3.506–36.158, *p* < 0.001) were independent risk factors.

**TABLE 2:** Logistic regression analysis for predicting PALN metastases.

Characteristics	Total (*n* = 329)	Univariate analysis		Multivariate analysis	
		OR (95% CI)	*p*	OR (95% CI)	*p*
Tumor size, cm					
≤ 5.3	131	1	< 0.001	1	0.002
> 5.3	198	5.412 (2.962–9.889)		3.129 (1.536–6.374)	
Tumor SUVmax					
≤ 9.8	229	1	0.012	1	0.360
> 9.8	100	2.436 (1.213–4.895)		1.481 (0.639–3.430)	
Bilateral PLN metastases					
No	209	1	< 0.001	1	0.091
Yes	120	16.282 (7.847–33.782)		2.411 (0.868–6.696)	
The number of positive PLN					
< 3	202	1	< 0.001	1	< 0.001
≥ 3	127	27.459 (11.342–66.477)		11.260 (3.506–36.158)	
Tumor stage					
T1b–T2	232	1	0.014	1	0.436
T3–T4	97	2.033 (1.154–3.581)		1.330 (0.649–2.727)	

Abbreviations: CI = confidence interval, OR = odds ratio, PALN = para‐aortic lymph node, PLN = pelvic lymph node, SUVmax = maximum standardized uptake value.

### 3.4 Construction and Evaluation of the Nomogram for the Prediction of PALN Metastases

**FIGURE 1:**
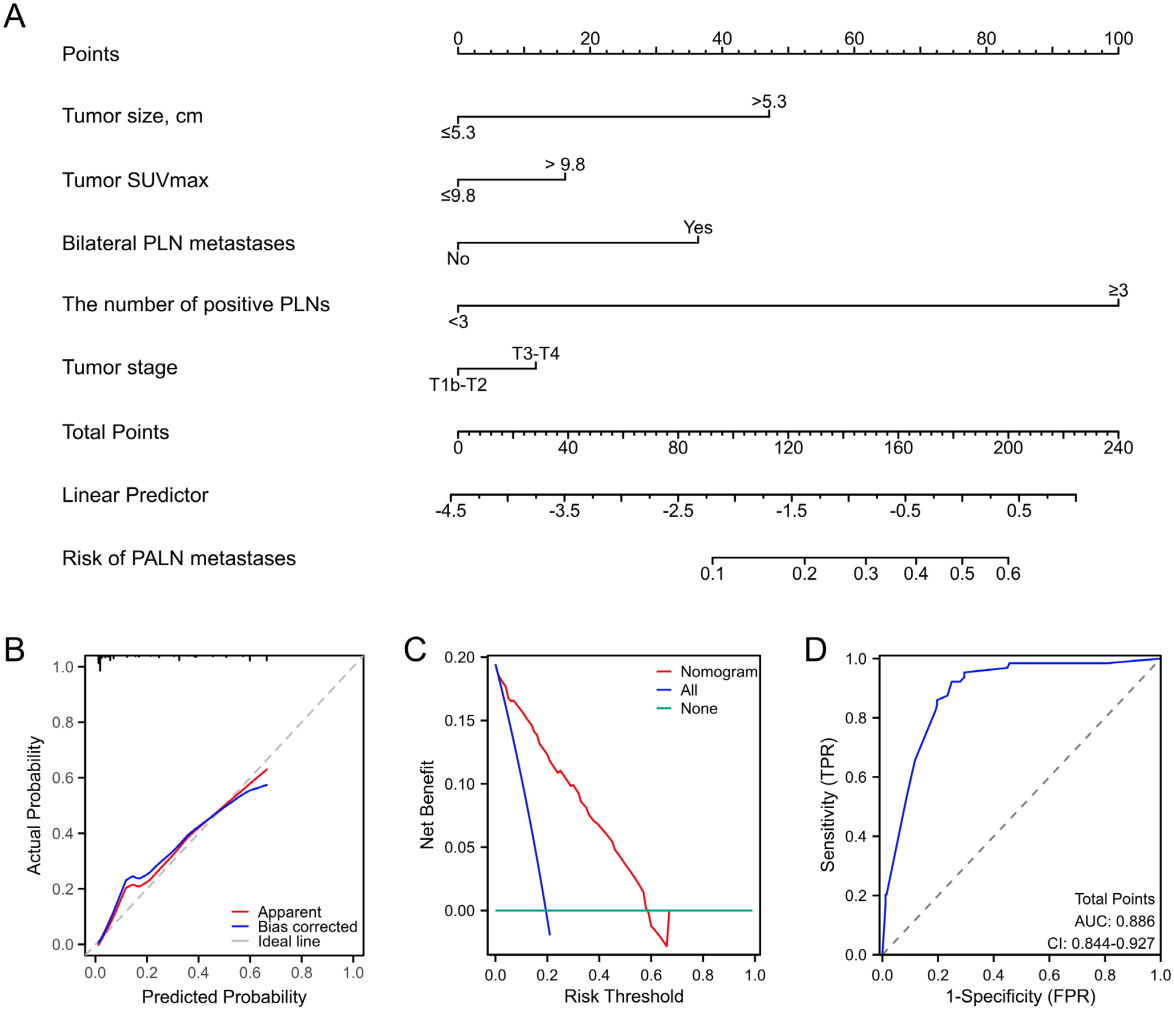
Nomogram (A). Calibration plot (B). DCA plot (C). ROC curve (D). DCA = decision curve analysis, PALN = para‐aortic lymph node, PLN = pelvic lymph node, ROC = receiver operating characteristic, SUVmax = maximum standardized uptake value.

A total of five covariates were included in the nomogram model to help quantify the risk of PALN metastases (Figure 1A). The number of positive PLNs ≥ 3 was the covariate with the highest effect in the model, which was converted into 100 points. When the total point was 148, it was predicted that the probability of PALN metastases was 0.30. The discriminatory ability (C‐index) of this model was 0.886 (95% CI = 0.844–0.927). The calibration plot demonstrated that the predicted probabilities of the nomogram agreed well with its actual probabilities (Figure 1B). DCA showed that the nomogram consistently performed better than the reference line across a broad range of probability thresholds, which indicated its clinical utility (Figure 1C).

The optimal cut‐off value of the nomogram was determined as 77.5. The AUC of the nomogram model was 0.886 (95% CI = 0.844–0.927), which showed that the nomogram had a good prediction effect in PALN metastases (Figure 1D). At the optimal cut‐off value, the nomogram had sensitivity, specificity, and positive and negative predictive values of 92.2%, 74.7%, 46.8%, and 97.5%, respectively.

### 3.5 Interpretation and Application of the Nomogram

Five variables were included in the nomogram model. The most significant factor was the number of metastatic PLNs ≥ 3, with a maximum score of 100 points. Patients with fewer than three metastatic PLNs were scored 0. The remaining variables were scored proportionally based on their effect size. The total score corresponded to the predicted probability of PALN metastasis. For example, a total score of 148 was associated with a 30% probability of PALN metastasis.

The nomogram allows for an individualized predictions of PALN metastasis before treatment based on five clinical features. For instance, a patient with cervical cancer presenting with a tumor max length of 6 cm, tumor SUVmax of 10, stage T4, bilateral PLN metastasis on PET/CT, and three metastatic PLNs, had a total score of 236.5 (tumor length > 5.3 cm: 42.5 points; SUVmax > 9.8: 17 points; bilateral PLN metastasis: 34.5 points; ≥ 3 metastatic PLNs: 100 points; stages T3–T4: 42.5 points). The model predicted a 79.76% probability of PALN metastasis.

## 4 Discussion

The involvement of PALNs is closely tied to large primary tumor size [15, 16, 17, 18, 19, 20]. It is generally believed that the risk of PALN metastases increases with the growth of tumor size. This study identified tumor size as an independent risk factor for PALN metastases and further delineated a size greater than 5.3 cm as a significant risk factor. Tumor size > 5.3 cm was further identified as an independent risk factor. The variance in specific data compared to other studies might stem from different methodologies in the measurement of tumor size. In this research, tumor diameter was measured through pelvic MRI or CT, which contrasted with Wang et al. [16]. They measured primary tumor size by gynecologic examination and found that tumor size ≥ 6 cm was a significant predictive factor of PALN metastases. Similarly, Zou et al. [20] based on surgical staging results, discovered that tumor size ≥ 4.8 cm was a predictive factor. The gynecologic examination of tumor size may overestimate tumor as it is based on a subjective assessment rather than an exact measurement [21]. The gynecologic examination was not as precise as the measurement using imaging or pathology, especially in the case of vaginal involvement and larger tumor diameter [22]. The detailed data from the study of Zou et al. remain unpublished. Due to their reliance on surgical staging for tumor diameter measurement, their sample skewed toward patients with stages IB3 and IIA2, which potentially did not represent the broader spectrum of LACC effectively. Moreover, the association between vaginal or parametrial involvement and PALN metastases was not found. T3–T4 stages were observed to be correlated with PALN metastases. However, the multivariate analysis showed no statistically significant association. These findings are similar to previous research that primarily focused on the relationship of the advanced FIGO stage with PALN metastases [16, 17, 18].

The presence of PLN metastases also significantly influences PALN metastases [17, 18, 20, 22, 23, 24, 25]. It was found that bilateral PLN metastases and the number of positive PLNs were associated with PALN metastases. After a multivariate logistic analysis, the number of PLNs ≥ 3 was found to be an independent risk factor of PALN metastases. Some previous studies have shown that PLN metastases were an independent risk factor for PALN metastases [17, 22, 24]. Ayhan et al. [25] reviewed 522 patients who suffered from 2009 FIGO stages IB1 to IIA2 cervical cancer and had undergone radical hysterectomy with pelvic and para‐aortic lymphadenectomy. They found that the number of PLNs ≥ 2 was an independent risk factor of PALN metastases. Moreover, Wang et al. [16] and Zou et al. [20] suggested that bilateral PLN metastases were a significant predictive factor. The results of this study were similar to those found. Differently, previous studies predominantly focused on FIGO 2009 stages IA to IIB, as these early stages are more frequently associated with surgical intervention. It was reported that the incidence of PALN involvement varies according to 2009 FIGO stages: 2%–5%, 10%–20%, 9%, 13%–30%, and 50% for stages IB, IIA, IIB, III, and IV, respectively [26]. Therefore, previous studies have typically encompassed only a small percentage of patients with PALN metastasis. As a consequence, this research enriches the dataset by focusing on a broader spectrum of LACC cases. The findings align with those of earlier studies.

The SUVmax of the cervical tumor at diagnosis acts as a risk factor for PALN metastases in LACC patients. PET/CT is recommended before treatment in patients with LACC to exclude lymph node and distant metastases. Among various quantitative parameters analyzed in PET/CT, SUVmax reflects the maximum uptake of the imaging agent in the target tissue. Higher SUVmax values indicate increased metabolic activity in tumors, which may correlate with more aggressive tumor behaviors. Previous studies have suggested that SUVmax is a sensitive biomarker for treatment response and prognosis in patients with cervical cancer [27], but few have investigated its association with PALN metastasis. This study aimed to evaluate tumor SUVmax as a potential predictor of PALN metastases. The group with PALN metastases was observed to have higher tumor SUVmax values compared to that without metastasis. Furthermore, a multivariate analysis was conducted, and it was determined that SUVmax > 9.8 was a significant risk factor for PALN metastases. Onal et al. [28] also reported higher tumor SUVmax in patients with metastases in both pelvic and para‐aortic areas. Considering the occurrence of PALN metastases without PLN involvement, PLN SUVmax was not included in this analysis. The role of tumor SUVmax in predicting PALN metastases warrants further investigation to substantiate this conclusion.

A nomogram was established to make an individualized prediction of PALN metastases and help to identify and stratify high‐risk patients. Tumor size > 5.3 cm, tumor SUVmax > 9.8, bilateral PLN metastases, the number of positive PLNs ≥ 3 and T3–T4 stages were combined in this nomogram. In previous studies, Shim et al. and Wang et al. also developed models to predict PALN metastases. Shim et al. [17] analyzed 245 LACC patients who underwent para‐aortic lymphadenectomy and observed that tumor size upon MRI and PALN status on PET/CT independently predicted PALN metastases. After a multivariate logistic analysis, a predictive scoring system was constructed. However, most patients in this study were stage IB2 or IIB 2009 FIGO (117/245 (47.8%) or 96/245 (39.2%), respectively) because one of the inclusion criteria was the pathologically confirmed PALN metastasis. Patients with more advanced diseases were excluded. Wang et al. [16] analyzed 1903 stages IA to IVA cervical cancer patients, among whom 130 (6.8%) suffered from PALN metastases. Multivariate analysis showed that non‐squamous cell carcinoma histology, bilateral PLN metastases, common iliac lymph node metastases, PLN convergence or muscle involvement (*p* < 0.001 for both) and tumor size ≥ 6 cm (*p* = 0.010) were significant predictive factors. Then, they developed a nomogram for the prediction of PALN metastases including these five factors. Nevertheless, the diagnosis of PALN and PLN metastases in some patients was determined by MRI, which may overlook occult metastatic lymph nodes. Additionally, the primary tumor size was measured by gynecologic examination, which was insufficiently accurate compared with imaging or pathology. This study therefore improves the shortcomings of the above two forecasting models. In this research, PET/CT was employed to evaluate lymph node involvement and an imaging technique was utilized to quantify tumor size. Based on the nomogram model, the number of positive PLNs ≥ 3 was a highly significant risk factor. In patients at high risk of PALN metastases, especially those with a tumor size > 5.3 cm and ≥ 3 positive PLN metastases identified on PET/CT, the system predicts approximately a 30% probability of PALN metastases. In such cases, prophylactic EFRT may offer benefits by enhancing the control of the para‐aortic region and improving overall survival.

The clinical significance of this study is multifaceted. Currently, prospective studies on prophylactic EFRT are limited. This study provides valuable insights and ideas for the design of future prospective studies. In situations where the indications for EFRT remain unclear, the findings of this study can serve as a guide for clinical decision‐making since the parameters of the model are clinically accessible. However, this study is subject to several limitations. One primary limitation is its single‐center design, which potentially leads to selection bias. To enhance the robustness and applicability of the nomogram, future research should involve external validation across multiple centers. However, the diagnostic accuracy of PET/CT in detecting PALN metastases remains limited compared with pathological assessment, particularly in identifying small‐volume micrometastases that fall below PET imaging resolution [29, 30]. Additionally, a retrospective cohort design was employed in this study. It introduced potential biases like selection and recall biases, as well as confounding variables that may influence the accuracy of the results. To obtain more precise cancer risk estimates, further prospective studies will be required.

## 5 Conclusion

It is found that tumor size > 5.3 cm and the number of positive PLNs ≥ 3 are independent risk factors for PALN metastases. A nomogram exhibiting pretty good accuracy in predicting the PALN metastases of LACC patients is established. For the improvement of therapeutic outcomes, prophylactic EFRT may help patients who are very likely to have PALN metastases. Specifically, patients with tumor size > 5.3 cm and positive PLNs ≥ 3 are most likely to gain from prophylactic EFRT.

## Author Contributions

**Tinglu Wang:** conceptualization (equal), data curation (equal), formal analysis (equal), methodology (equal), visualization (equal), writing – original draft (lead). **Jinchen Wei:** data curation (equal), formal analysis (equal), writing – original draft (equal). **Li Jiang:** formal analysis (equal), visualization (equal), writing – original draft (equal). **Lulu Huang:** formal analysis (equal), writing – original draft (equal). **Tingting Huang:** validation (equal), writing – original draft (equal). **Shanshan Ma:** project administration (equal), visualization (equal), writing – review and editing (equal). **Qiufeng Huang:** data curation (equal), writing – original draft. **Yong Zhang:** conceptualization (equal), data curation (equal), supervision (equal), writing – review and editing (equal). **Fang Wu:** conceptualization (equal), data curation (equal), funding acquisition (equal), methodology (equal), writing – review and editing (equal).

## Ethics Statement

This study was conducted in accordance with the Declaration of Helsinki, and approved by the Ethics Committee of the First Affiliated Hospital of Guangxi Medical University (protocol code 2023‐E323‐01 and date of approval 2023‐07‐05).

## Conflicts of Interest

The authors declare no conflicts of interest.

## Data Availability

This study is reported as per the Transparent Reporting of a Multivariable Prediction Model for Individual Prognosis or Diagnosis guidelines. In accordance with the journal's guidelines, we will provide our data for independent analysis by a selected team by the Editorial Team for the purposes of additional data analysis or for the reproducibility of this study in other centers if such is requested.
